# Editorial: Foundation models for robot design

**DOI:** 10.3389/frobt.2026.1927761

**Published:** 2026-09-10

**Authors:** Mahdi Tavakoli, Emmanuel Benjamin Vander Poorten, Sanja Dogramadzi, Jay Carriere, Amir Hooshiar, Arash Arami, Hamidreza Kasaei

**Affiliations:** 1 Department of Electrical and Computer Engineering, University of Alberta, Edmonton, AB, Canada; 2 Department of Mechanical Engineering, KU Leuven, Leuven, Belgium; 3 School of Electrical and Electronic Engineering, University of Sheffield, Sheffield, United Kingdom; 4 Department of Electrical and Software Engineering, University of Calgary, Calgary, AB, Canada; 5 Department of Surgery, McGill University, Montreal, QC, Canada; 6 Department of Mechanical and Mechatronics Engineering, University of Waterloo, Waterloo, ON, Canada; 7 Department of Artificial Intelligence, University of Groningen, Groningen, Netherlands

**Keywords:** embodied AI, foundation models, large language models, robot design, task planning, vision-language-action models

Foundation models are beginning to change not only how robots interpret language, but also how robotic systems are designed, adapted, evaluated, and deployed. In robotics, their promise lies in connecting high-level human intent with perception, decision-making, control, and physical embodiment. Yet this promise also exposes a central design challenge: general-purpose models must be integrated into systems constrained by real-time control, safety, embodiment, hardware variability, and the uncertainty and structure of the physical world. The articles in this Research Topic examine this challenge from complementary perspectives, spanning task planning, human-model interfaces, perception-driven task identification, motion generation, and physics-informed model adaptation.

A recurring theme is that foundation models are most useful in robotics when embedded within structured architectures rather than treated as unconstrained black boxes. In Borate et al. present a hierarchical framework in which large language models support high-level task interpretation, task allocation, and event-driven replanning for heterogeneous robot teams. The work highlights an important direction for robot design: LLMs can provide flexible reasoning and natural-language interfaces, but their usefulness depends on explicit representations of robot capabilities, task status, events, and available low-level skills. The architecture, therefore, places the LLM within a larger deliberation-execution loop, with robot-level modules responsible for executable skills and low-level control.

The importance of interface design is further emphasized by Tuladhar and Kinney-Lang treat prompt framing not merely as wording, but as a measurable system parameter in hybrid LLM-reinforcement learning control. Their advisor-arbiter testbed shows that a hybrid LLM + RL agent can outperform RL-only and LLM-only baselines under constrained interaction budgets, while ablation experiments indicate that structured advisor information contributes beyond the presence of additional text. Different prompt framings also produce heterogeneous effects. This suggests that prompts, personas, and relational framing should be evaluated as consequential design variables in robotic systems, particularly when language models participate in multi-step decision making.

Another contribution addresses how foundation models can help robots decide what is worth acting on. In Martinson et al. combine open-vocabulary perception, scene reconstruction, change detection, and model-based validation to identify candidate tasks for domestic service robots. The key insight is that “change affords action”: rather than treating every detectable object as a possible target, the robot can focus on objects that have changed relative to a baseline and then reason about whether intervention is appropriate. The work connects foundation models to situated usefulness, showing how perception and reasoning can be organized around environmental context and human preferences.

At the motion-generation level, by Shi et al. addresses a practical bottleneck in embodied AI: diffusion-based policies can represent multimodal action distributions, but iterative denoising can be too slow for real-time robotic control. FRMD accelerates the action-generation module using movement-primitive parameterization and trajectory-level consistency distillation, reducing inference latency while maintaining task performance on manipulation benchmarks. By accelerating the motion-generation component without changing upstream vision or language encoders, the work illustrates that foundation-model-based robot design must account for computational timing, not only model capability.

Finally, by Wang et al. broadens the discussion beyond language and vision-language-action models. The authors investigate how a physics-informed hybrid model for pneumatic artificial muscles can be adapted across different actuator entities using a physically motivated adapter. Although the work is not a foundation model in the large-scale pretraining sense, it addresses a closely related principle: reusable representations become valuable when they can be adapted efficiently to new embodied systems while retaining physical structure. This is particularly relevant for soft, wearable, and bio-inspired robots, where embodiment varies substantially and purely data-driven retraining can be costly.

Taken together, the articles suggest that foundation models for robot design should be understood as components of hybrid, embodied, and task-aware systems. Their value lies not only in scale but also in how they are connected to structured priors, physical constraints, perception pipelines, control policies, and human intent. The contributions also point to several open questions: how to benchmark foundation-model-enabled robotic systems across changing environments; how to improve safety and reliability when language-based reasoning affects action; how to manage latency and computational cost; how to adapt models across robot morphologies and materials; and how to design human-facing interfaces that are both expressive and predictable.

The next phase of research in this area will likely move from demonstrating that foundation models can be used in robotics to determining how they should be designed into robotic systems. This requires careful attention to architecture, evaluation, embodiment, and responsible deployment. The papers collected here provide early but concrete steps toward that goal, showing that the design of future robots may increasingly depend on the design of the interfaces, priors, representations, and adaptation mechanisms through which foundation models are grounded in the physical world.

